# A retrospective study of mortality for perioperative cardiac arrests toward a personalized treatment

**DOI:** 10.1038/s41598-022-17916-3

**Published:** 2022-08-12

**Authors:** Huijie Shang, Qinjun Chu, Muhuo Ji, Jin Guo, Haotian Ye, Shasha Zheng, Jianjun Yang

**Affiliations:** 1 Department of Anesthesiology, Pain and Perioperative Medicine, The First Affiliated Hospital of Zhengzhou University, No. 1 Jianshe East Road, Zhengzhou, 450000 Henan China; 2 Academy of Medical Sciences, Zhengzhou University, Zhengzhou, Henan China; 3 Department of Anesthesiology and Perioperative Medicine, Zhengzhou Center Hospital Affiliated to Zhengzhou University, Zhengzhou, Henan China; 4 Department of Anesthesiology, The Second Affiliated Hospital of Nanjing Medical University, Nanjing, Jiangsu China; 5 Department of Oncology, Shanxi Province Hospital of Traditional Chinese Medicine, Taiyuan, Shanxi China

**Keywords:** Computational biology and bioinformatics, Diseases, Health care, Medical research, Risk factors

## Abstract

Perioperative cardiac arrest (POCA) is associated with a high mortality rate. This work aimed to study its prognostic factors for risk mitigation by means of care management and planning. A database of 380,919 surgeries was reviewed, and 150 POCAs were curated. The main outcome was mortality prior to hospital discharge. Patient demographic, medical history, and clinical characteristics (anesthesia and surgery) were the main features. Six machine learning (ML) algorithms, including LR, SVC, RF, GBM, AdaBoost, and VotingClassifier, were explored. The last algorithm was an ensemble of the first five algorithms. k-fold cross-validation and bootstrapping minimized the prediction bias and variance, respectively. Explainers (SHAP and LIME) were used to interpret the predictions. The ensemble provided the most accurate and robust predictions (AUC = 0.90 [95% CI, 0.78–0.98]) across various age groups. The risk factors were identified by order of importance. Surprisingly, the comorbidity of hypertension was found to have a protective effect on survival, which was reported by a recent study for the first time to our knowledge. The validated ensemble classifier in aid of the explainers improved the predictive differentiation, thereby deepening our understanding of POCA prognostication. It offers a holistic model-based approach for personalized anesthesia and surgical treatment.

## Introduction

Perioperative cardiac arrest (POCA) is a rare but extremely serious risk event with high mortality during anesthesia and surgery. It is commonly defined as the loss of circulation that prompts resuscitation with chest compressions and/or defibrillation in the operating room^1,2^. The reported incidence of anesthesia-related POCA ranges from 0.04 to 8 per 10,000 administered anesthetics, and it is associated with high immediate mortality rates varying between 20 and 60%^3–7^. Accurately predicting survival and promptly making correct decisions pose a huge challenge for anesthesiologists and clinicians under uncertain and dynamic environments.

The incidence and causes of cardiac arrests related to anesthesia have been studied over the last two decades^1^. Nevertheless, understanding of POCA and controlling the related risk factors are still in their infancy. Several studies^8–11^ analyzed individual variables associated with the survival of cardiac arrests by meta-analysis. The main issue with this approach is that effects are often multivariate rather than univariate, making results prone to bias. Multiple disease severity scores predicting survival have been developed as a tool for risk stratification after cardiac arrest^12–21^; however, as they usually have suboptimal predictive accuracy for a specific patient population, they should be cautiously extrapolated and applied to an individual patient in hospital.

Recently, machine learning has emerged as an effective approach to integrate multiple quantitative variables to improve accuracy of incidence predictions in medicine, with the potential to dramatically improve healthcare delivery^22–26^. Specifically, in the fields of anesthesiology and cardiac arrest research, it has recently been shown that ML is a promising method for a more comprehensive understanding of the risk factors and a supporting tool for healthcare improvement^27–31^.

Therefore, this study reported all cardiac arrests that occurred in a surgical population pre-intra-post anesthesia in one of the largest Chinese tertiary hospitals during an 8-year period, and examined causes of mortality with ML in addition to the univariate method of ANOVA. After validating the ML models, they can be used to identify the mortality risk factors and predict survival outcome of an individual patient. The data bring more information about anesthesia and surgery in addition to patients’ demographic characteristics, and ML models may help offer more potential to understand and manage the procedure than traditional resuscitation algorithms^33^. This study may provide a basis for designing model-based prediction and care management strategies of anesthesia/surgery to improve the prognosis and survival of POCA. The aim of this retrospective study was to identify factors of POCA and help to improvement in the prevention and management of POCA. This work will open up an avenue for a personalized anesthesia and surgery strategy, with a better treatment and a higher survival rate attained.

## Methods

### Data collection

This retrospective study was approved by Human Research Ethics Committee of the First Affiliated Hospital of Zhengzhou University (number: KY-2021-0084). The study was registered at the Chinese Clinical Trial Registry (ChiCTR2100051737). The requirement for obtaining a written informed consent from patients was waived due to the retrospective nature of the study. The study was performed in accordance with the principles of the Declaration of Helsinki. Electronic medical records of 380,919 patients who had undergone a surgical procedure between December 2012 and June 2020 were reviewed by three of the authors (Huijie Shang, Qinjun Chu, Jin Guo) in July 2020. Brain-dead organ donors and babies undergoing cardiac compressions due to arrest immediately after caesarean section were excluded from the analysis. Patients on cardiopulmonary bypass or extracorporeal membrane oxygenation were also excluded because cardiac compressions are not needed in such situations and the use of such devices can significantly affect clinical outcomes. The “perioperative” period was defined as the time from entering the operating room to exiting the postanesthesia care unit. Cardiac arrest was defined as any condition that required performing chest compressions or defibrillation. From the anesthetic records, 150 patients who suffered POCA with a full record were selected for this study. Data were classified into patient demographic characteristics, operation-related variables, and cardiac arrest–related variables. The patients’ demographic characteristics included gender, age, body mass index (BMI), comorbidities, emergency, trauma, and five-category physical status by ASA PS. The operation-related variables comprised anesthetic type, surgical type, operative position, the amount of blood lost, blood transfused anti-arrhythmic drug use, and continuous infusion of vasoactive drugs. Cardiac arrest–related variables included arrest cause, arrest time, whether or not defibrillation was done, and duration of CPR. The primary outcome was in-hospital mortality of POCA patients until hospital discharge.

### Statistical analysis

The patients’ characteristics were compared by mortality outcomes. The statistical methods used in this work were the same as those used in a previous study^31^. The analyses were done in R programming language, version 3.6.1. The code has been uploaded (refer to Supplementary Document 1.2 in Online Supplementary Materials).

### Machine learning models

Six algorithms were explored. Five of them, namely LR, SVC, RF, GBM, and AdaBoost, are the most commonly used algorithms for binary classification problems in medicine^37^. An additional one is an ensemble approach, which is realized through a voting classifier aggregating the prediction of multiple classifiers. Therefore, we designed VotingClassifier, which combines the predictions of the aforementioned five models to improve prediction robustness.

Notably, it was quite a challenge to obtain a robust and accurate ML model given that the data were scarce because POCA is a very rare event. A thorough effort was made in this work as follows.

1. A five-fold cross-validation resampling procedure was used to evaluate the models on the limited training data to reduce the prediction bias. The bootstrapping method was further leveraged to minimize the potentially large prediction variance. For each fold, we extracted the true positive rate and false positive rate and calculated the area under the receiver operating characteristic curve (AUC), the mean of which was used as the optimization metric. Based on this series of results, we obtained a confidence interval of AUC to show the robustness of an ML classifier.
2. Grid and random hyperparameter search were used to search for optimal hyperparameters.

### Model explainability

The ML models except LR are all “black-box” algorithms. To break down the black box, we employed several model-agnostic methods, including (1) Permutation feature importance to globally understand the importance and effects of features; (2) SHAP to calculate local feature importance for every observation^38^; and (3) LIME to analyze individual predictions (accumulated local effects)^39^. All ML analyses were conducted using open-source software libraries of Python, version 3.7.3.

## Results

### Patients’ characteristics and statistical analysis

There were 380,919 patients who had undergone a surgical procedure, with 163 POCAs, of which 13 were excluded and 150 included (Supplementary Fig. 2). As shown in Table 1, 150 POCA patients were investigated. A total of 81 patients died prior to hospital discharge, resulting in a survival rate of 46%. The average age was 49.4 (± 18.5) years, with 96 (64.0%) patients being male and 73 (48.7%) being emergency cases. A total of 145 (96.7%) patients underwent general anesthesia, and 91 (60.7%) patients were in ASA PS III–V. Fourteen patients experienced cardiac arrest during induction, while 13 patients experienced cardiac arrest during intubation. The majority of cardiac arrests (*N* = 102; 68.0%) occurred during surgery. The common causes of POCA were preoperative complications (*N* = 34; 22.7%), related to anesthesia (*N* = 23; 15.3%), and surgical complications (*N* = 41; 27.3%).

**Table 1:** Patient demographics and operative variables of entire cohort stratified by survival to hospital discharge.

	All patients	Survived to hospital discharge		*p*-Value
		Yes	No	
Number of patients (%)	150 (100.0)	69 (46.0)	81 (54.0)	
**Gender, N (%)**				0.016
Female	54 (36.0)	32 (46.4)	22 (27.2)	
Male	96 (64.0)	36 (57.2)	60 (74.1)	
Age, years (SD)	49.4 (18.5)	51.3 (19.4)	47.1 (17.2)	0.155
BMI, kg/m^2^ (SD)	24.3 (3.9)	24.6 (4.1)	23.8 (3.7)	0.233
**Comorbidities and medical history, N (%)**				
Diabetes	10 (6.7)	7 (10.1)	3 (3.7)	0.186
Hypertension	43 (28.7)	24 (34.8)	19 (23.5)	0.146
Cardiac disease	34 (22.7)	15 (21.7)	19 (23.5)	1.000
Pulmonary disease	36 (24.0)	14 (20.3)	22 (27.2)	0.489
Hepatic disease	14 (9.3)	4 (5.8)	10 (12.3)	0.298
Renal disease	20 (13.3)	9 (13.0)	11 (13.6)	1.000
Neurological disease	31 (20.7)	13 (18.8)	18 (22.2)	0.823
Cancer	33 (22.0)	15 (21.7)	18 (22.2)	1.000
**Surgical type, N (%)**				0.020
Abdominal	63 (42.0)	28 (40.6)	35 (43.2)	
Neurosurgery	17 (11.3)	3 (4.3)	14 (17.3)	
Thoracic	37 (24.7)	15 (21.7)	22 (27.2)	
Throat	12 (8.0)	8 (11.6)	4 (4.9)	
Others	21 (14.0)	14 (20.3)	7 (8.6)	
Emergency, *N* (%)	73 (48.7)	24 (34.8)	49 (60.5)	0.005
Trauma, *N* (%)	19 (12.7)	11 (15.9)	8 (9.9)	0.352
**Anesthetic type, N (%)**				1.000
General	145 (96.7)	66 (95.7)	79 (97.5)	
Local	5 (3.3)	2 (2.9)	3 (3.7)	
**Operative position (%)**				0.016
Lateral decubitus	21 (14.0)	13 (19.1)	8 (9.8)	
Lithotomy	4 (2.7)	4 (5.9)	0 (0.0)	
Prone	3 (2.0)	3 (4.4)	0 (0.0)	
Supine	122 (81.3)	48 (70.6)	74 (90.2)	
**ASA PS, N (%)**				0.000
1	4 (2.7)	4 (5.8)	0 (0.0)	
2	55 (36.7)	35 (50.7)	20 (24.7)	
3	37 (24.7)	16 (23.2)	21 (25.9)	
4	36 (24.0)	12 (17.4)	24 (21.0)	
5	18 (12.0)	1 (1.4)	17 (24.6)	
**Arrest time, N (%)**				0.937
Induction	14 (9.3)	7 (10.1)	7 (8.6)	
Intubation	13 (8.7)	5 (7.2)	8 (9.9)	
Surgery	102 (68.0)	46 (66.7)	56 (69.1)	
NA*	21 (14.0)	10 (14.5)	11 (13.6)	
Defibrillate, *N* (%)	72 (48.0)	30 (43.5)	42 (51.9)	0.482
**Arrest cause, N (%)**				0.200
Anesthesia	23 (15.3)	11 (15.9)	12 (14.8)	
Comorbidities	34 (22.7)	10 (14.5)	24 (29.6)	
Surgery	41 (27.3)	20 (29.0)	21 (25.9)	
Unknown	52 (34.7)	27 (39.1)	25 (30.9)	
Hemorrhage, median [Q1, Q3] (ml)	100.0 [2.3, 500.0]	95.0 [4.3, 200.0]	200.0 [0.8, 1075.0]	0.032
Blood transfusion, median [Q1, Q3] (ml)	0.0 [0.0, 1000.0]	0.0 [0.0, 0.0]	0.0 [0.0, 1712.0]	0.002
Epinephrine, median [Q1, Q3] (mg)	2.0 [0.1, 5.9]	0.5 [0.0, 2.0]	4.0 [2.0, 7.9]	0.000
Atropine, median [Q1, Q3] (mg)	0.0 [0.0, 0.5]	0.0 [0.0, 0.5]	0.0 [0.0, 0.5]	0.737
Amiodarone, median [Q1, Q3] (g)	0.0 [0.0, 0.0]	0.0 [0.0, 0.0]	0.0 [0.0, 0.0]	0.598
Ephedrine, median [Q1, Q3] (mg)	0.0 [0.0, 0.0]	0.0 [0.0, 2.3]	0.0 [0.0, 0.0]	0.182
Methoxamine, median [Q1, Q3] (mg)	0.0 [0.0, 0.0]	0.0 [0.0, 0.0]	0.0 [0.0,0.0]	0.885
CPR, median [Q1, Q3] (min)	30.0 [10.0, 37.0]	11.0 [1.0, 37.0]	37.0 [27.0, 43.0]	0.000

NA*: not available.

The following variables were significantly different between the survivor and non-survivor groups (*P* < 0.05): gender, surgical type, emergency, operative position, ASA PS, hemorrhage, blood transfusion, epinephrine, and CPR. Accordingly, the favorable categories for survival were female sex, throat (or other) surgery, non-emergency, and ASA PS I–II. In contrast, there was a higher probability of mortality in male individuals, neurosurgery, emergency, supine operative position, massive hemorrhage and blood transfusion, and ASA PS V. A higher epinephrine dose (4.0 [IQR 2.0–7.9] versus 0.5 [IQR 0.0–2.0] mg) was administered, and a longer CPR (37.0 [IQR 27.0–43.0] versus 11.0 [IQR 1.0–37.0] min) was performed during cardiac arrest in non-survivors.

Other variables, such as age, BMI, trauma, arrest time, use of defibrillation, and arrest cause, were not significantly different between the two groups. There was no evidence that the administration of drugs except epinephrine was directly associated with survival or death.

In addition, comorbidities and medical history were generally not strongly associated with mortality. The observed difference in the presence of hypertension (*P* = 0.146) between the survival and death groups was 14.6%, which indicates that hypertension might be a remarkably influential comorbidity for further exploration.

### ML models

The 150 patients were split into two subgroups in a gender-stratified manner, i.e., 112 (75%) and 38 (25%) for training and testing of the ML models, respectively. To preserve the same gender proportions of patients in each subgroup as in the total patients, the data were split in a gender-stratified manner. The predicting outcome was the probability of mortality.

**Figure 1:**
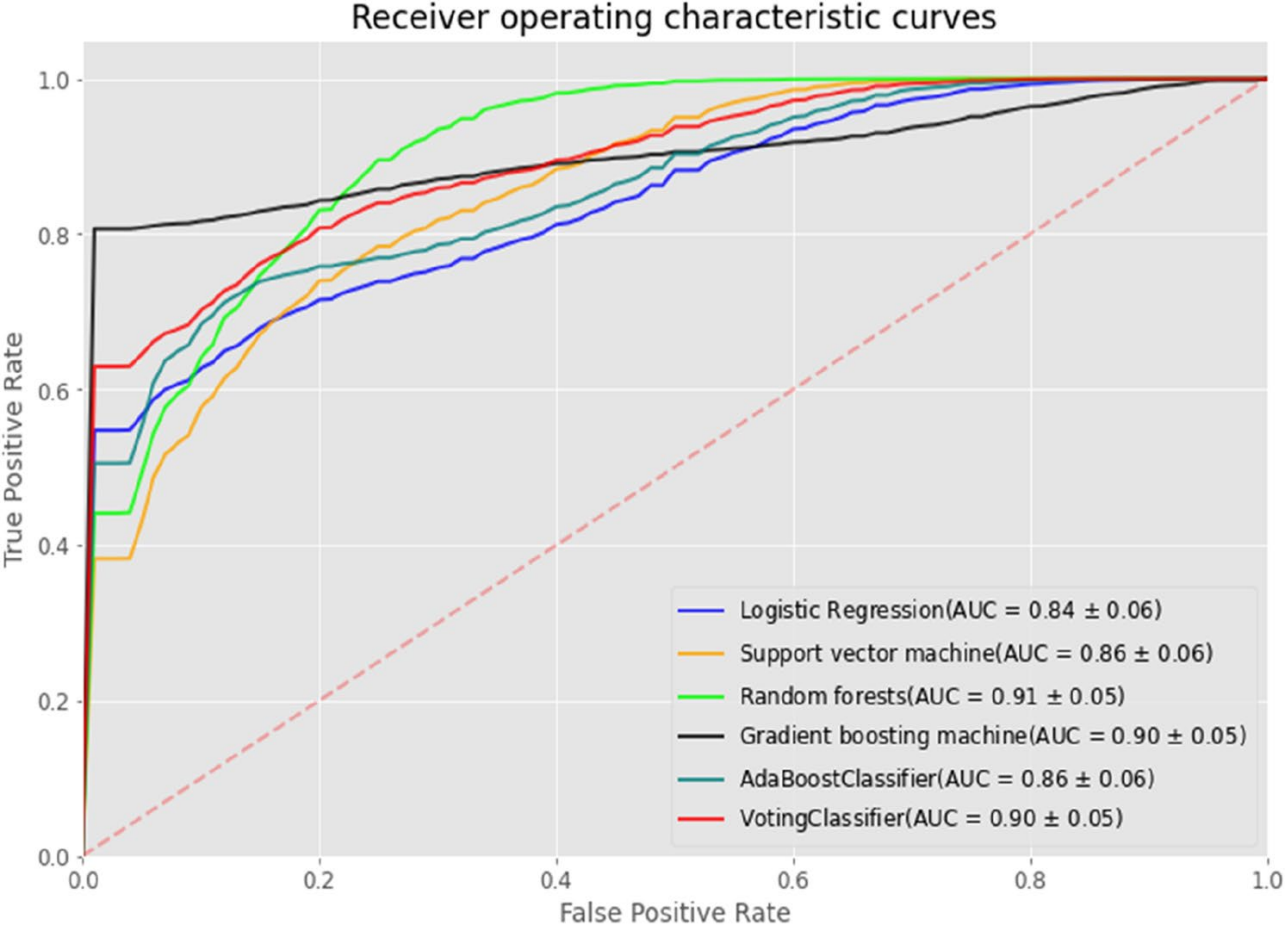
ROC curves for the six ML models on the test data. The AUC value of each model is represented by “(AUC = mean ± standard deviation)”, which was estimated from 1000 bootstrap resamples of predictions on the test data. Each ROC curve is visualized by corresponding plot with shaded bands.

Figure 1 shows the Receiver Operating Characteristic (ROC) curves generated with the test data by the six ML models, including LR, SVC, RF, GBM, AdaBoost, and VotingClassifier, and their AUCs were 0.84, 0.87, 0.91, 0.90, 0.87, and 0.90, respectively.

In binary classification, the most basic metric/bench-mark is the confusion matrix given that “accuracy,” “precision,” “recall,” “f1-score,” “ROC,” and “AUC” all stem from the confusion matrix^38^. We used these multiperspective performance measures to fairly judge the predictive models.

As shown in Table 2, three significantly accurate ML models were the RF (AUC, 0.91 [95% CI, 0.79–0.98]), the ensemble (AUC, 0.90 [95% CI, 0.78–0.98]), and the GBM (AUC, 0.90 [95% CI, 0.79–0.98]). It is not a surprise that as a simple and interpretable classifier, the LR produced the poorest accuracy (AUC, 0.84 [95% CI, 0.71–0.95]). Taking other metrics into account, it was demonstrated that the VotingClassifier outperformed all of the other classifiers, with the highest values of accuracy (0.84), precision (0.85), recall (0.85), and f1-score (0.85).

**Table 2:** Performance of the six ML models for the estimation of mortality of patients with a POCA.

Models	AUC [95%CI]	Accuracy	Precision	Recall	f1-score
Logistic regression	0.84 [0.71–0.95]	0.74	0.78	0.70	0.74
Support vector classifier	0.87 [0.73–0.96]	0.79	0.83	0.75	0.79
Random forest	0.91 [0.79–0.98]	0.82	0.84	0.80	0.82
Gradient boost machine	0.90 [0.79–0.98]	0.82	0.84	0.80	0.82
Adaptive boosting classifier	0.87 [0.73–0.97]	0.76	0.79	0.75	0.77
Ensemble (VotingClassifer)	0.90 [0.78–0.98]	0.84	0.85	0.85	0.85

The 95% CI of AUC was calculated from 1000 bootstrap resamples of predictions on the test data.

**Figure 2:**
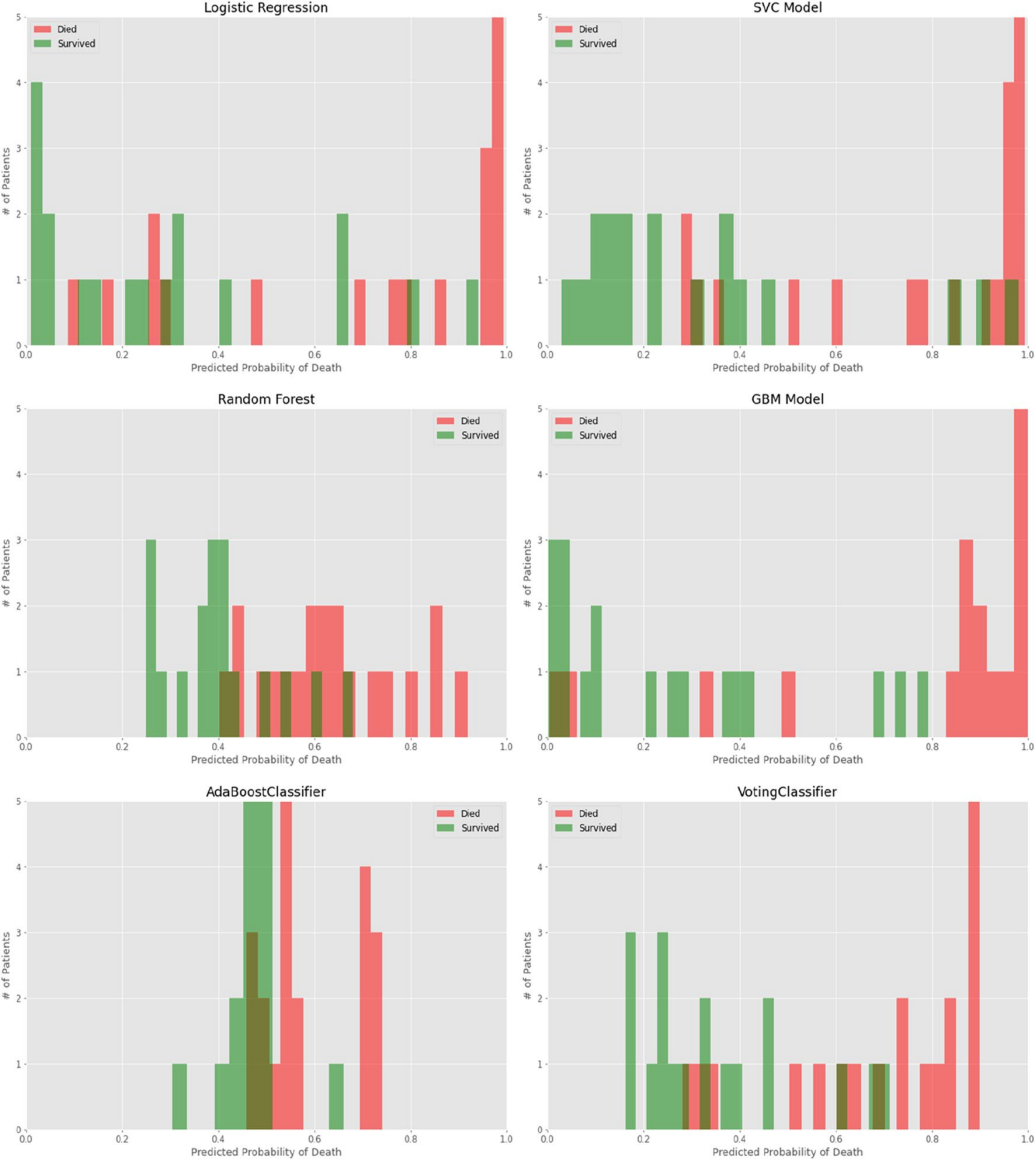
Probability curves for each ML model. Survivors indicated in green, and non-survivors in red. *p* < 0.005 for ensemble versus other models.

**Figure 3:**
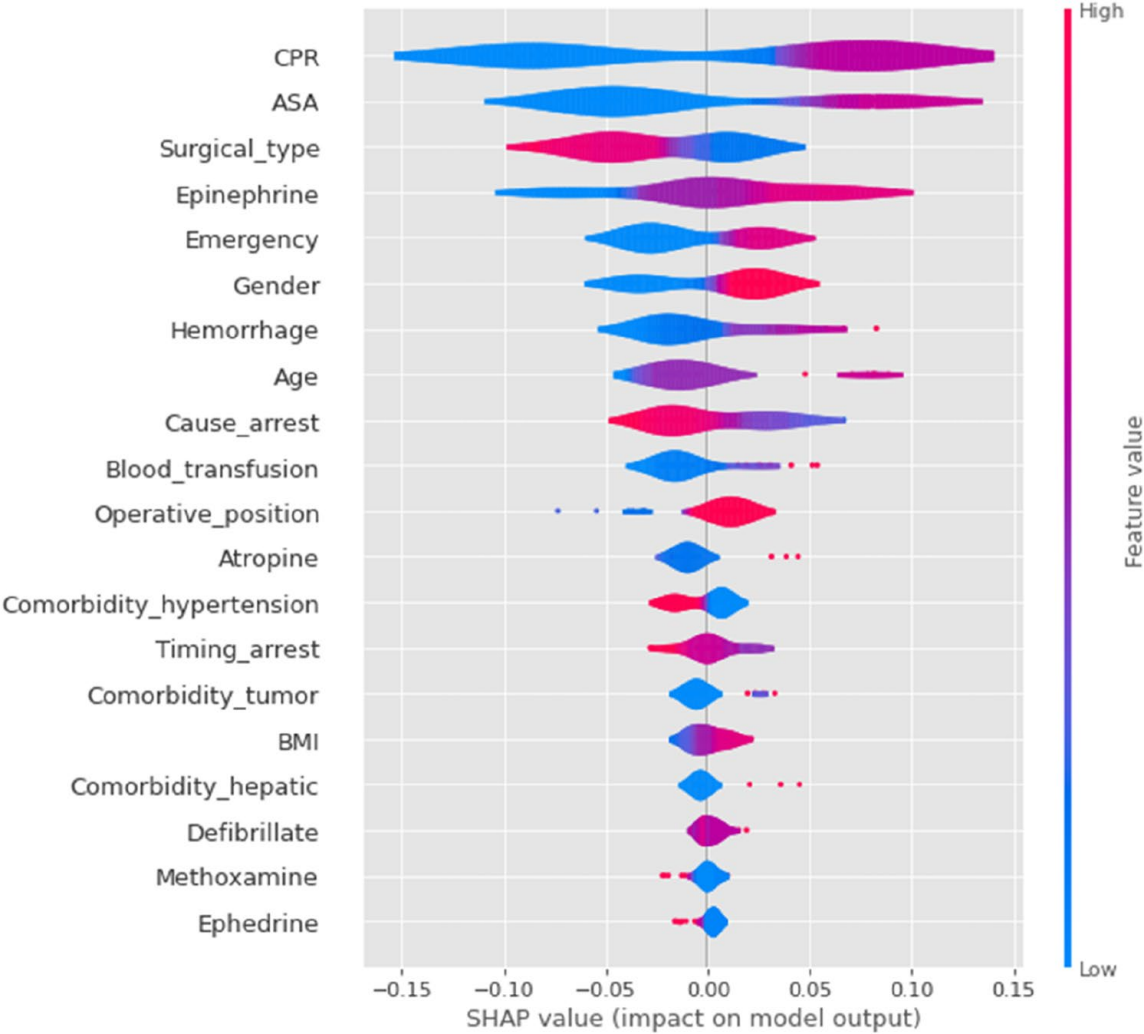
SHAP importance plots of the mortality and risk factors for the ensemble ML model (VotingClassifier). The features are ranked by importance. Each row represents the impact of a feature on the outcome of mortality, with higher SHAP values indicating higher likelihood of a positive outcome. For a binary feature, like gender, “male” → “1” is shown in red while “female” → “0” is shown in blue. For the detailed mapping of categorical features, please refer to the code online (such as “ < 12 ys” → “0”, “12 ~ 40 ys” → “1”, “40 ~ 65 ys” → “2”, “ > 65 ys” → “3”).

We further considered two aspects to analyze the prediction performance of the models. One was probability curves for each ML model (Fig. 2); another was model comparisons with respect to mortality estimation across age groups (Fig. 3).

As shown in Fig. 2, the LR estimated a higher probability of survival. Corresponding to a threshold of 50%, the false negative (FN) of mortality was 6, and the false positive (FP) was 4; this means that six patients who died were wrongly classified into survivors, while four patients who survived were wrongly predicted to have died. For the SVC model, FN was 5 and FP was 3, with low variance in probability attributed to all survivors. For the RF and the GBM, the misclassified values were smaller, i.e., FN = 4 and FP = 3. The VotingClassifer brought about the smallest misclassifications, with FN = 3 and FP = 3. In addition, the GBM and VotingClassifier demonstrated significant separation of the dead individuals from the survivors, with lower overlap between the two groups.

As shown in Supplementary Fig. 1, the VotingClassifier was the best classifier for age groups “ < 12 years” and “ ≥ 65 years”, and probably the second best for age groups “12–40 years” and “40–65 years” (outmatched only by the RF). The GBM model tended to significantly overestimate mortality in age groups “ < 12 years” and “ ≥ 65 years”.

To summarize, the ensemble ML model (VotingClassifier) outperformed all of the other classifiers by making better predictions and achieving better performance than any single contributing model. Moreover, it reduced the spread or dispersion of the predictions with higher robustness.

### Model explainability

First, we applied the SHAP to explain predictions on the test data by the VotingClassifier. The SHAP summary, combining feature importance with feature effects, was visualized with violin plots to present the distribution of Shapley values (Fig. 3). The position on the y-axis was determined by the feature and that on the x-axis by the Shapley value.

The following results were obtained, and most of them enhanced the previous ANOVA analyses:

1. A high mortality risk was strongly associated with the top 10 important features, in the following order of importance: longer CPR (≥ 60 min), higher ASA PS (IV–V), surgical type (“abdominal” or “neurosurgery”), higher dose of epinephrine (> 6 mg), emergency, male sex, massive hemorrhage (≥ 800 mL), older age (especially > 65 years), cause of arrest (“anesthesia” or “comorbidities”), or massive blood transfusion (≥ 800 mL);
2. In the less important features, operative position (“supine”), arrest time (“induction”), comorbidity (“cancer” or “hepatic disease”), BMI (“obese”), and atropine (> 0.65 mg) showed slight positive associations with mortality;
3. Counterintuitively but interestingly, the comorbidity of hypertension appeared to have a protective effect on survival prior to hospital discharge, similar as recently reported^33^.

All effects described the model behavior and were not necessarily causal in the real world, which was why we used the term “association” rather than “causation” in the above statements^32^.

**Figure 4:**
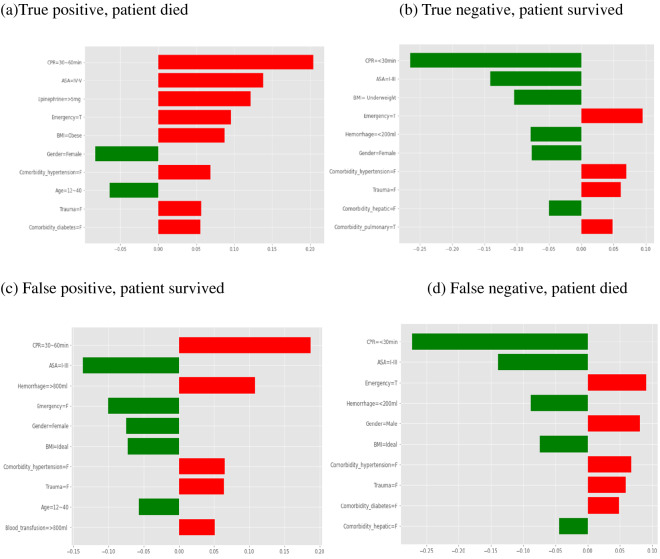
LIME explainer for four typical scenarios. (**a**) True positive, patient died, i.e., a correctly classified non-survivor, (**b**) True negative, patient survived, i.e., a correctly classified survivor, (**c**) False positive, patient survived, i.e., an incorrectly classified survivor (predicted to die), and (**d**) False negative, patient died, i.e., an incorrectly classified non-survivor (predicted to survive). Features with a green bar favored survival, and those with a red bar were predictive of mortality. The x-axis shows how much each feature added to or subtracted from the final probability value for the patient. Each weight can be interpreted in the context of the original probability; if a feature is absent for a patient, it can be numerically added to or subtracted directly from the initial probability.

Second, we interpreted the VotingClassifier with the LIME explainer, particularly to explore misclassification of prediction. Four typical cases corresponding to the four quadrants in a confusion matrix (TP, TN, FP, FN), were compared. The top 10 features are presented in Fig. 4, with the weight of each feature represented in either green or red depending on whether it favored survival or death, respectively.

In Fig. 4a, we show a specific individual with a high probability of mortality (80%). This patient died as predicted, and the key risk-associated factors were longer CPR (30–60 min, ~ 20% impact on mortality), ASA PS of IV–V (~ 14% impact), epinephrine > 5 mg (~ 12% impact), emergency (~ 9% impact), obesity (~ 8% impact), and no hypertension (~ 7% impact). In one TN case (Fig. 4b), the predicted probability of mortality was 24%. The patient actually survived and was correctly predicted. The survival-favorable features were CPR ≤ 30 min (~ 27% increased probability of survival), ASA PS of I–III (~ 14% increase), underweight BMI, hemorrhage < 200 mL, female sex, and no hepatic disease.

In one FP case (Fig. 4c), the predicted probability of mortality was 62%, but the patient survived. The key unfavorable features for survival were CPR 30–60 min (~ 19% impact) and hemorrhage ≥ 800 mL (~ 12% impact), to which the misclassification could be attributed. In one FN case (Fig. 4d), the predicted probability of mortality was 39%. However, the patient died. The most survival-favorable feature was CPR ≤ 30 min (~ − 28% impact), which probably overpowered other survival-unfavorable features, such as emergency, thereby leading to this misclassification.

## Discussion

Given the fact that POCA is a quite rare incidence, it is hard to access to an abundant amount of data. Moreover, there are few papers about cardiac arrests in China. The present study investigated POCA in 380,919 patients at a Chinese tertiary hospital. Overall, the incidence of POCA was 3.9 per 10,000 surgical procedures with a mortality of 54% prior to hospital discharge. All of the ML models used in this study, except for the LR, are “black-box” algorithms, which provide great accuracy at the cost of low interpretability^33^. There are multiple dangers of a decision made by ML without opening the black box, as follows: (1) It is usually hard to explain the predictions to clinicians, which is a barrier to the adoption of ML for high stakes decisions^35^; (2) More and more concerns or regulations specific to ML have been emerging on interpretability and its predictive reasoning (for example, the EU General Data Protection Regulation).

First, a global model-agnostic method of permutation feature importance was employed in this work. The results were not shown in this article because some evident drawbacks of this method were found: (1) shuffling the feature added randomness and the results usually varied greatly; (2) some features were inherently correlated, and this method was very biased by unrealistic data instances.

In this study, SHAP and LIME were demonstrated to be two competent local model-agnostic methods in the model explainability. Instead of calibrating global feature contributions, these two methods train local surrogate models to explain individual predictions with more solid insights generated, such as how to rank a feature by importance with a favorable or unfavorable impact value on prediction outcome. We obtained contrastive explanations with the two explainers, particularly to explore misclassification, making the ensemble ML model more transparent and shedding light on their applications in clinical decision-making. Explanations can be used to interrogate and rectify the ensemble model when such a misclassification surfaces.

Our study has several limitations. First, although the ultimately validated ensemble model was robust and accurate, the size of data used was still relatively small. Specifically, there were only eight patients younger than 12 years, which was probably why most of the ML models (except the ensemble) failed to satisfactorily predict the outcome of this age group. In the future, more internal data and even external data may bring more benefits to establish generalizability and further increase the model fidelity. Second, our dataset had no information on post-arrest care and discharge disposition. Thus, it was impossible to systematically follow up and assess long-term recovery and survival of the discharged patients. Third, our study had a single-center retrospective design, and our dataset was abstracted from the electronic medical records by the researchers in this study, who had not been involved in the clinical treatment of the patients; therefore, the accuracy of the dataset was verified.

Finally, an ML model is not a “magic button,” although it would have reached a “super-human” performance. Like most ML approaches, the ML models validated in this study focused on predicting outcomes rather than on understanding causality, i.e., they found correlations but not causation. As an example, it was revealed in this study that two top predictors of risk for in-hospital mortality were CPR and epinephrine. The ensemble model predicted that longer CPR and higher dose of epinephrine were associated with a higher probability of death. In fact, the opposite was true; namely, patients (with severe ASA PS or massive hemorrhage) would be at a higher risk for serious complications and sequelae, even mortality, if insufficient CPR and/or epinephrine treatment were not timely delivered.

In clinical practice, accurate prediction models allow for improved medical prognostication, earlier identification of patients at high risk of complications, better risk adjustment and utilization of critical care resources, and more effective patient-physician-family communication.

In this study, the validated ensemble model provides superior prediction accuracy by virtue of high fidelity to data across various age groups and high robustness to uncertainty, as well as good discrimination between survivors and non-survivors. The data comprised operative parameters in addition to patients’ demographic characteristics, which makes it possible to integrate operational optimization and/or tactical planning with the model by managing the operative parameters and procedure.

One application scenario is early recognition of problems and suggestion of actions to avoid critical events. For an individual patient, an optimal combination of anesthesia management, surgical type, operative position (if optional), and treatment drugs could lead to a significantly improved probability of survival until hospital discharge (some exploratory simulations were done but not shown in this article). Another application scenario is that the model could enhance rational patient risk monitoring during operations, with drug doses administered in a timely fashion (target-controlled infusion), resulting in precision, efficacy, and safety of intravenous anesthesia delivery. In short, this model-based optimization opens an avenue for a personalized anesthesia and surgery strategy, with a better treatment and a higher survival rate attained.

Furthermore, clinicians hesitate to apply a black-box algorithm that is hard for them to understand and trust^33,34^. In this work, the explainers (LIME and SHAP) may pinpoint logics of decision-making and mitigate issue of clinical liability, encouraging clinicians to understand and leverage ML to assist decision-making and change management in practice.

## Conclusion

The ensemble ML model makes solid predictions of mortality on the data of POCA patients’ demographic and operative parameters, bringing a more comprehensive understanding of the risk factors and patient prognostics prior to hospital discharge, compared to the approach with ANOVA. Furthermore, the explainers of LIME and SHAP provide a more comprehensible and holistic approach to the assessment of prognosis of an individual patient. All of these results may assist risk management of in-hospital cardiac arrest with improved patient-centered and personalized care.

## Supplementary Information

Supplementary Information.

## Data Availability

The data and codes for analysis are accessible on *GitHub* if required, at https://github.com/niuneo/Risk-factor-analysis-of-mortality-for-perioperative-cardiac-arrest-using-machine-learning*.*

## Abbreviations

POCA: Perioperative cardiac arrest
ML: Machine learning
SHAP: Shapley additive Explanations
LIME: Local interpretable model-agnostic explanations
ANOVA: Analysis of variance
BMI: Body mass index
CPR: Cardiopulmonary resuscitation
ASA PS: American society of anesthesiologist’s physical status classification
LR: Logistic regression
SVC: Support vector classifier
RF: Random forest
GBM: Gradient boosted machine
AdaBoost: Adaptive boosting classifier
AUC: Area under the receiver operating characteristic curve
ROC: Receiver operating characteristic
