# Supplementary material for: A retrospective study of mortality for perioperative cardiac arrests toward a personalized treatment
Source: Sci Rep. 2022 Aug 12;12:13709. doi: 10.1038/s41598-022-17916-3 (PMC9374678; doi:10.1038/s41598-022-17916-3)
Supplement: Supplementary file 1 — Supplementary Information. [file 41598_2022_17916_MOESM1_ESM.docx]

**Online Supplementary Materials**

Shang H, Chu Q, et al. A retrospective study of mortality for perioperative cardiac arrests towards personalized treatment

**1.** **The data and codes for analysis**, including the following files,

1.1. The data file: “*150CA_EN20210701.xlsx*”;

1.2. The R code for statistical analysis: “*ANOVA-test_patient-characteristics.Rmd*”;

1.3. The Python code for ML model validations: “*Predicting CA Mortality V4.ipynb*”;

1.4. The python code for ML model explainability: “*Model Explanation_V4.ipynb*”.

**2.** **Supplementary Figure1**. Predicted Mortality Ratio by Age Group. Sensitivity analysis comparing model performance across age groups by six machine learning models

**1.** **The data and codes for analysis**, are accessible on *Github* if required, at

[*https://github.com/niuneo/Risk-factor-analysis-of-mortality-for-perioperative-cardiac-arrest-using-machine-learning*](https://github.com/niuneo/Risk-factor-analysis-of-mortality-for-perioperative-cardiac-arrest-using-machine-learning)*,* including the following files,

1.1. The data file: “*150CA_EN20210701.xlsx*”;

1.2. The R code for statistical analysis: “*ANOVA-test_patient-characteristics.Rmd*”;

1.3. The Python code for ML model validations: “*Predicting CA Mortality V4.ipynb*”;

1.4. The python code for ML model explainability: “*Model Explanation_V4.ipynb*”.

**2.** **Supplementary Figure 1.**
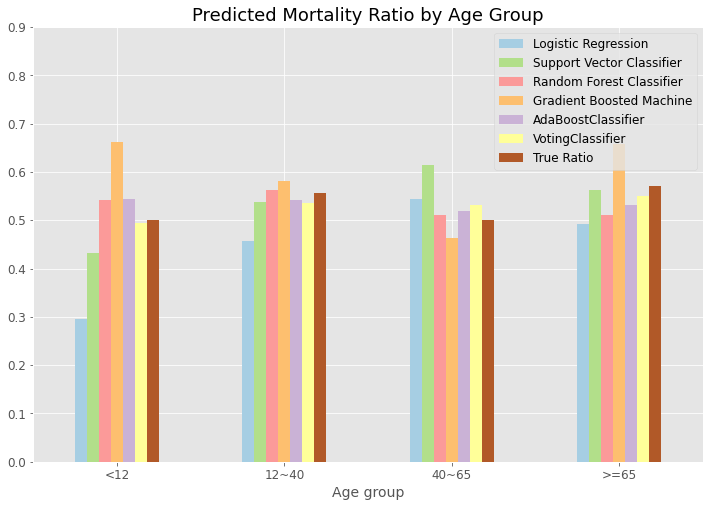


**Supplementary Figure 1.** Sensitivity analysis comparing model performance across age groups**.** True mortality for each group is indicated in dark red.

**Exclusions**

13 patients

**380,919 patients reviewed**

(December 2012—June 2020)

**Perioperative cardiac arrests**

163 patients

**Included for analysis**

150 patients

**Survivors**

69 patients

**Non-survivors**

81 patients

**Supplementary Figure 2.** Study flow diagram.
